# Use of Intravascular Micro-Axial Left Ventricular Assist Devices as a Bridging Strategy for Cardiogenic Shock: Mid-Term Outcomes

**DOI:** 10.3390/jcm13226804

**Published:** 2024-11-12

**Authors:** Balakrishnan Mahesh, Prasanth Peddaayyavarla, Kenny Nguyen, Aditya Mahesh, Corrine Corrina Hartford, Robert Devich, Gianna Dafflisio, Nandini Nair, Miriam Freundt, Robert Dowling, Behzad Soleimani

**Affiliations:** 1 Heart and Vascular Institute, Pennsylvania State Milton S Hershey Medical Center, Hershey, PA 17033, USA; 2 NHS Arden & Greater East Midlands Commissioning Support Unit, St John’s House, Leicester LE1 6NB, UK; 3 College of Medicine, Pennsylvania State University, University Park, PA 16802, USA; 4 Christ Hospital, Cincinnati, OH 45219, USA

**Keywords:** ECMO, cardiogenic shock, micro-axial left ventricular assist device, temporary mechanical circulatory support, heart recovery, heart replacement

## Abstract

**Objectives**: Patients in cardiogenic shock (CS) may be successfully bridged using intravascular micro-axial left ventricular assist devices (M-LVADs) for recovery or determination of definitive therapy. **Methods**: One hundred and seven CS patients implanted with M-LVADs from January 2020 to May 2024 were divided into four groups; group-1: 34 patients (transplant); group-2: 25 patients (LVAD); group-3: 42 patients (postcardiotomy CS (PCCS)); group-4: 6 patients (decision/recovery but excluded from analysis). Multivariable logistic regression and Multivariable Coxregression models identified predictors of early -hospital and late mortality, and Odds ratios (ORs) and hazard ratios (HRs) with *p* < 0.05, respectively, were considered statistically significant. SPSS 29.0 and Python 3.11.1. were used for analyses. **Results**: Complications included device-malfunction (6%), gastrointestinal bleed (9%), long-term hemodialysis (21%), axillary hematoma requiring re-exploration (10%), heparin-induced thrombocytopenia (4%) requiring heparin therapy cessation/initiation of argatroban infusion, and non-fatal stroke (11%). Early hospital mortality included 13 patients: 2 in group-1, 1 in group-2, 10 in group-3 (*p* = 0.02). In the Logistic-Regression model, category of CS requiring an M-LVAD was significant (OR = 4.7, *p* = 0.05). Patients were followed for 4.5 years (mean follow-up was 23 ± 17 months), and 23 deaths occurred; group-1: 3 patients, group-2: 5 patients, and group-3: 15 patients (*p* = 0.019). At 4.5 years, actuarial survival was 90.7 ± 5.1% in group-1, 79.2 ± 8.3% in group-2, 62.8 ± 7.7% in group-3 (*p* = 0.01). In the Cox-Regression model, M-LVAD category (HR = 3.63, *p* = 0.04), and long-term postoperative dialysis (HR = 3.9, *p* = 0.002) emerged as predictors of long-term mortality. **Conclusions**: In cardiogenic shock, mid-term outcomes demonstrate good survival with M-LVADs as bridge to transplant/durable LVADs and reasonable survival with M-LVADs as a bridge to recovery following cardiotomy, accompanied by reduced ECMO usage, and early ambulation/rehabilitation.

## 1. Introduction

Cardiogenic shock (CS) in adults has a high mortality, especially when occurring postcardiotomy (PCCS). In the setting of acute heart failure emerging as a major threat to survival, the use of temporary mechanical circulatory support (MCS) is becoming the mainstay in stabilizing patients experiencing cardiogenic shock. With the evolution of the field of temporary mechanical circulatory support (t MCS) many newer devices have been developed of which micro-axial circulatory pumps seem to be very effective in unloading the left ventricle, augmenting cardiac output/cardiac power/the mean arterial pressure (MAP), increasing coronary and systemic perfusion pressures, and optimizing pulmonary hemodynamics and right ventricular function [1,2,3,4,5]. These newer devices have surpassed the role of extra corporeal membrane oxygenator [ECMO] in PCCS, which is associated with a high mortality of 42–44% [6,7]. A more contemporary series described better results with ECMO in PCCS of up to 67%, though that relied on the use of levosimendan, which is not FDA-approved in the USA [8]. Micro-axial ambulatory LVADs (M-LVADs) may provide better support in patients with cardiogenic shock who may be eligible for a transplant or a durable left ventricular assist device (LVAD) or recover [9]. This approach can maintain or improve end-organ function and enables independent ambulation, thereby preserving muscle mass and potentially improving outcomes after operative therapy. This study was undertaken to prospectively assess outcomes in cardiogenic shock patients receiving M-LVADs as a bridge to advanced surgical therapies or as a bridge to decision and/or recovery.

## 2. Methods

Between January 2020 and May 2024, 107 patients underwent placement of M-LVADs for cardiogenic shock and followed till September 2024. These included: group-1—transplant: 34 patients (32%) patients underwent cardiac transplantation with one of these patients receiving a heart and kidney transplant; group-2—LVAD: 25 patients (23%) underwent placement of a durable LVAD; group-3—PCCS: 42 patients (39%) had placement of an M-LVAD as a bridge to recovery from PCCS, of which 16 patients had ECMO initially and 20 patients had an M-LVAD straightaway for weaning from cardiopulmonary bypass (CPB) following cardiotomy. In 6 patients (4.3%) it was implanted as a bridge to recovery/decision (Graphical abstract). Four patients were deemed to be ineligible for either transplantation or an LVAD due to social circumstances (*n* = 3) or metastatic cancer (*n* = 1). All these patients subsequently died following explantation of the M-LVAD. The fifth patient was a Jehovah’s Witness and demanded transfer to a center experienced in implanting LVADs in Jehovah’s Witnesses and was lost to follow-up. The sixth patient had biopsy-proven giant-cell myocarditis that improved with immunosuppressive therapy and the device was explanted on the 28th day. These 6 patients were excluded from analysis. In group-1 and group-2, the cardiogenic shock was due to decompensation against a background of chronic heart failure due to ischemic or non-ischemic pathologies. Patients presenting with cardiogenic shock following a STEMI were treated by an interventional cardiologist with a smaller M-LVAD providing 3–3.5 L/min of flow and placed via the femoral artery. These patients did not fall into group-1–3 based on our classification, as none of these patients was bridged with the larger M-LVADs placed surgically either to recovery or transplant/durable LVAD therapy. Therefore, they were outside the scope of this study.

The primary study end point was in-hospital mortality, and the secondary end points were overall survival and device-related complications, including implantation site bleeding, device failure requiring exchange, hemolysis, dialysis, or stroke at any time. Data were collected prospectively and entered into a database maintained on secure hospital servers and analyzed retrospectively (STUDY00017861). All patients were followed up throughout the study until the end of the study period or death, and there were no missing patients. This study was approved by the institutional review board of Penn State University, approval included a waiver of informed consent. All relevant data are within the manuscript and its Supporting Information files. SPSS 29.0 (IBM Inc., Armonk, NY, USA) and Python 3.11.1 were used for statistical analyses

### 2.1. Predictors of In-Hospital Mortality

Normally distributed data are reported as means and standard deviations, using the analysis of variance test for differences between groups. *p* < 0.05 denotes statistical significance. Variables with *p* < 0.1 were then entered into a multivariable logistic regression model to identify predictors of early hospital mortality, and the data are reported as odds ratios (ORs). *p* < 0.05 denotes statistical significance. 

### 2.2. Assumption Check for Multivriable Logistic Regression Model

Prior to fitting the model, we examined the assumptions underlying logistic regression. To check linearity between independent variables and log-odds, we conducted visual inspection using partial regression plots for the continuous independent variables age and Body mass index [BMI]. For both variables, slight deviation from linearity was observed, suggesting a nuanced association with the outcome. We assessed multicollinearity assumption through the variance inflation factor (VIF). Age and BMI have high VIFs, indicative of multicollinearity concerns. We further inspected the predictive power of these two variables on the independent variable using kernel density estimate plots. Both variables showed little discriminative power. We therefore removed the two variables from the final model. To evaluate the independence assumption, we plotted residual deviance against observation order and discerned no systematic patterns, reaffirming the independence of residuals. 

### 2.3. Variable Selection

We also employed recursive feature elimination (RFE) with a random forest classifier to identify the most relevant features for predicting our target variable. We chose this technique to reduce potential for the model to overfit the data as it considers the contribution of each independent variable to the model’s performance. After applying RFE to our dataset, we identified the variables that exhibited the strongest predictive power. Overall, utilization of RFE underscores our commitment to employing rigorous and principled methodologies in feature selection, ultimately enhancing the robustness and reliability of our predictive modeling approach.

For the internal validation of the fitted model, we employed a 7-fold cross-validation technique. We chose this technique due to having a relatively small sample size. The area under the receiver operating characteristic curve (AUC) was utilized as the primary metric for evaluating model performance. Notably, the best-performing model achieved an AUC of 0.598, indicative of its limited ability to discriminate between different classes with a satisfactory level of accuracy.

### 2.4. Predictors of Long-Term Survival

Survival data were plotted by the Kaplan–Meier method, using the log-rank test for differences between the groups. Variables with *p* < 0.1 were then entered into a multivariable Cox regression model to identify predictors of late mortality, and the data were reported as hazard ratios (HRs). *p* < 0.05 denotes statistical significance. 

### 2.5. Assumption Check for Multivriable Cox Regression Model

We performed an assessment of the proportional hazards assumption for the Cox regression model, a prerequisite for valid interpretation of hazard ratios (HRs). The model included covariates identified in Kaplan–Meier analysis as potential predictors of survival with *p* < 0.1. The analysis involved scrutinizing Schoenfeld residuals and conducting the proportional hazard test. The M-LVAD category demonstrated low cardinality and, therefore, we opted for a strategic approach and decided to stratify the analysis by this particular variable. Stratification ensures a more nuanced exploration of the variable’s impact on survival by allowing the baseline hazard to vary within each stratum, yet preserving the impacts of covariates across all strata. This adaptive adjustment underlines our commitment to transparent and rigorous statistical methodologies.

### 2.6. Technique of M-LVAD Insertion

**Figure 1:**
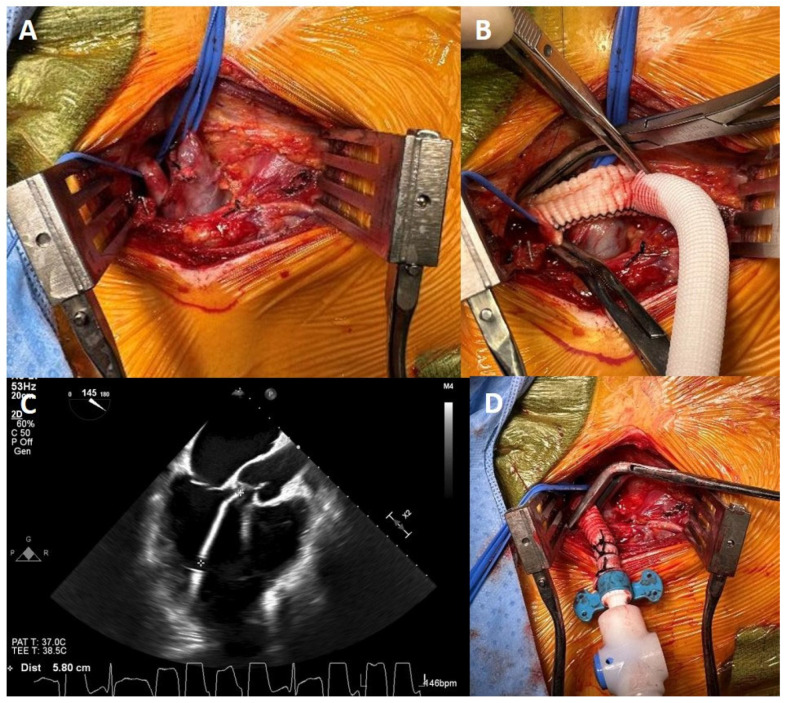
(**A**): Exposure of the axillary vessels. (**B**): Anastomosis of the graft to the axillary artery using 5-0 Prolene suture. (**C**): TEE confirming length and position of the M-LVAD inside the LV cavity. (**D**): Completed M-LVAD insertion.

A 7 cm transverse incision is made 2 cm inferior to the lateral half of the right clavicle. The pectoralis major and minor muscles are divided, and the axillary artery and vein are identified and mobilized, carefully avoiding the cords of the brachial plexus. Vessel loops are placed proximally and distally (Figure 1A). Frequently, the subclavian vein overlies the artery and is required to be mobilized to one side with vessel loops. The patient is then given 5000 units of heparin followed by the placement of a side-biting clamp on the subclavian artery. We prefer the subclavian artery to be >6.5 mm in size as measured by preoperative Doppler examination, for Impella-5.5 placement. A longitudinal arteriotomy is performed. An 8 mm Gelweave Dacron graft (Terumo Aortic US, Sunrise, FL, USA) is then beveled at a 45° angle and anastomosed end-to-side to the subclavian artery using a continuous 5-0 Prolene suture (Figure 1B). The obturator for the M-LVAD is then secured in place within the graft with the special plastic clips. A 0.035″ Rosen J-wire (Cook Inc., Bloomington, IN, USA) is used in conjunction with an AL-1 (Cordis Corporation, Miami Lakes, FL, USA) catheter to reach the aortic root. With the AL-1 catheter pointing towards the aortic valve, the 0.035” J-wire is used to cross the aortic valve, followed by the AL-1 catheter. The J-wire is then removed and a 0.018″ wire that is included with the M-LVAD is passed through the AL-1 catheter and positioned in the apex of the LV, and the AL-1 catheter is removed. The M-LVAD is then flushed with heparinized saline and backloaded onto the 0.018″ wire. The M-LVAD is passed over the 0.018” wire under fluoroscopic and trans-esophageal echocardiographic (TEE) guidance with appropriate position in the left ventricle (Video S1).

The guidewire is then removed and the M-LVAD support is initiated at 1 L/min and then gradually increased to 5.5 L/min. Appropriate position of the device is confirmed on TEE and fluoroscopy, with the tip positioned 5.5–5.8 cm below the aortic valve and pointing into the cavity of the LV and away from the interventricular septum, lateral wall, and the mitral valve (Figure 1C). The graft is then trimmed and secured to the obturator with multiple silk sutures, brought out through the lateral aspect of the incision, and secured in that position once again with multiple silk sutures. After ensuring adequate hemostasis, the incision is closed in a standard manner with interrupted braided absorbable sutures and skin staples (Figure 1D). 

Anticoagulation while on M-LVAD support is sodium bicarbonate solution in the purge solution and systemic heparin titrated to maintained a partial thromboplastin time (PTT) of 40–50 s. Patients diagnosed with heparin-induced thrombocytopenia (HIT) are treated with argatroban infusion to maintain a PTT of 40–50 s. 

## 3. Results

Mean age was 58.4 ± 13.2 years, and mean BMI was 29.9 ± 5.4 kg/m^2^; 14 patients were females (14%). Percutaneous right ventricular assist device (p-RVAD) support was required in 15 patients (15%): 5 patients (5%) after an LVAD, 10 patients (10%) for PCCS, where it was used in conjunction with an M-LVAD for LV support. No patients in the bridge to transplant group required p-RVAD support. Their preoperative characteristics are outlined in Table 1. Twenty patients were transitioned from ECMO to an M-LVAD: three patients (3%) in group-1, six patients (6%) in group-2, and eleven patients (11%) in group-3.

### 3.1. Postoperative Results

Complications are outlined in Table 2 and included device malfunction requiring exchange in 6 patients (6%), axillary hematoma re-exploration in 10 patients (10%), gastrointestinal bleed in 9 patients (9%), long-term hemodialysis in 21 patients (21%), heparin-induced thrombocytopenia in 4 patients (4%) requiring cessation of heparin therapy and commencement of argatroban infusion, and central nervous system (CNS) ischemic stroke in 11 patients (11%). For CNS strokes, we have an aggressive protocol in our hospital. The bedside nurse alerts to a ‘brain-attack’ upon noticing any CNS symptoms/signs, triggering an immediate response from neurology and a CT angiogram, and upon identification of thrombus involving a large cerebral vessel, it is promptly extracted by the interventional team. With this aggressive protocol in place, we noticed that eight patients had complete resolution of their neurological disabilities while three patients had residual deficits, isolated limb weakness (*n* = 2) and dysphasia (*n* = 1). None of these CNS events was life-threatening. Perioperative mortality included 13 patients (13%): 2 in group-1, 1 in group-2, and 10 in group-3 (*p* = 0.02).

### 3.2. Predictors of Perioperative Mortality

We fitted a multivariable logistic regression (LR) model to explore the association between the independent variables and hospital mortality. The category of cardiogenic shock requiring M-LVAD placement was statistically significant (OR = 4.7 (0.9–24), *p* = 0.05) (Table 3).

### 3.3. Long-Term Results

**Figure 2:**
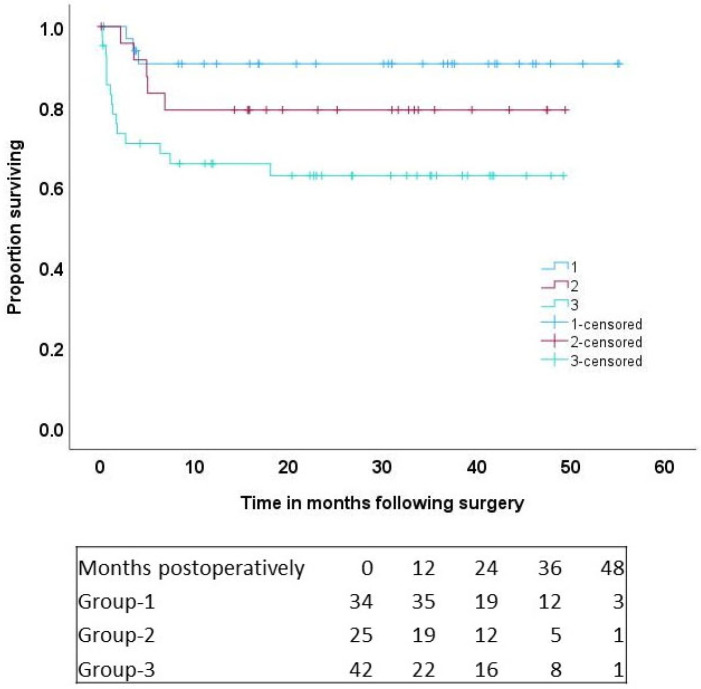
Survival by categories of Micro-axial left ventricular assist device support. Group-1, transplant; group-2, durable left ventricular assist device; group-3, postcardiotomy support.

Patients were followed for up to a maximum of 4.5 years; the mean follow-up period was 23 ± 17 months. During the follow-up period, 23 deaths occurred; group-1: 3 patients (8.8%), group-2: 5 patients (20%), and group-3: 15 patients (35.7%) (*p* = 0.019). At 4.5 years, actuarial survival was 90.7 ± 5.1% in group-1, 79.2 ± 8.3% in group-2, and 62.8 ± 7.7% in group-3 (*p* = 0.01, Figure 2).

### 3.4. Predictors of Long-Term Mortality

#### Cox Regression

Based on the univariate Kaplan–Meier survival analysis which identified M-LVAD category, long-term postoperative dialysis, and postoperative gastrointestinal bleeding as the predictors with *p* < 0.1, we conducted a Cox regression analysis to assess the impact of various covariates, making the appropriate statistical adjustments [10]. M-LVAD category (HR = 3.63 (1.03–12.9) *p* = 0.04) and long-term postoperative dialysis (HR = 3.9 (1.6–9) *p* = 0.002) emerged as statistically significant predictors of long-term mortality (Table 4).

## 4. Discussion

Cardiogenic shock (CS) in adults has a high mortality, especially when occurring following cardiotomy. Recent studies have evaluated the role of ECMO in PCCS and have described a high mortality of up to 42–56% [6,7,11]. A more contemporary series described improved results with ECMO in PCCS, though that relied on the use of levosimendan, which is not available for use in the United States of America [8]. Presently, ECMO is an accepted strategy to prolong survival in these patients [12]. 

Newer intravascular micro-axial LVADs appear to be suitable for bridging patients with CS to transplant or a durable LVAD [4] with/without a p-RVAD [13]. Placement of these upper-body devices facilitates weaning from femoral veno-arterial ECMO and therefore early ambulation and prehabilitation, thereby preserving muscle mass, making these patients better candidates for transplant or LVAD, and also reducing ECMO-related hematological, neurological, and limb complications. Assessment of RV function is difficult in patients on ECMO even in the presence of a plethora of measurements [14]; poor RV function adversely impacts post-LVAD survival. Similarly, assessment of pulmonary vascular resistance (PVR) is difficult in patients on ECMO [15]; high PVR can adversely impact posttransplant survival. Assessment of RV function and PVR can be easily accomplished with an M-LVAD, thereby greatly improving outcomes following transplant or LVAD implantation. Furthermore, ECMO has potentially deleterious effects on pulmonary, neurologic, and coagulation systems, along with limb ischemia and infective complications [16], all of which are improved by switching from ECMO to M-LVAD therapy.

Previously, Pawale et al. [17] described their results in 43 patients with refractory CS treated with direct implantation of durable LVADs without any bridging strategies. They described operative mortality of 14% and survival of 73.9% at 12 months and concluded that this strategy provides good mid-term outcomes and obviates the need for bridging strategies for CS, thereby avoiding associated complications including repeat surgeries for bleeding, requirement for LV venting, limb ischemia, temporary devices, and/or cannula exchanges. Our results have shown better actuarial survival of those bridged to an LVAD with micro-axial temporary devices of 79.2 ± 8.3% at 4.5 years. Furthermore, direct implantation without a bridging strategy does not allow for time to consider the implantation of such an expensive device; some patients may not be LVAD candidates due to lack of social support, disseminated malignancy, or active gastrointestinal lesions predisposing to profuse bleeding on anticoagulation. Some patients may be better served by cardiac transplantation; some patients may recover following percutaneous coronary intervention for acute MI and therefore not require a durable LVAD or transplant, and some patients may indeed have a potentially treatable condition [18]. In our series, we excluded five patients from advanced therapies due to the aforementioned considerations. The micro-axial temporary LVADs allow time for LV recovery or development of the most suitable long-term treatment plan.

In our series of patients, we were able to demonstrate low rates of adverse events combined with good survival by utilizing these micro-axial temporary LVADs. Using these devices for bridging patients with cardiogenic shock, we were able to triage them into categories of transplantation, durable LVAD support, or anticipated recovery and also avoid complications described in previous studies with other more invasive temporary support devices [17,18]. Importantly, in patients who were eligible for cardiac transplantation, implantation of these M-LVADs enabled early mobilization and ambulation as status-2 on the waiting list, thereby preserving muscle mass. In the transplant cohort, we demonstrated an excellent actuarial survival of 90.7 ± 5.1 at 4.5 years.

Our study is also one of the larger ones to describe postcardiotomy support in 42 patients using the M-LVAD. In 16 patients, ECMO was used to wean from cardiopulmonary bypass (CPB); within 1 week, these patients were transitioned to micro-axial LVAD support as ECMO-weaning echocardiographic studies determined that longer duration of LV support was required. p-RVAD support was also required in four patients. In 26 patients, micro-axial LVAD was used to wean from CPB. Six patients also required percutaneous RVAD. Actuarial survival was 62.8% ± 7.7 at 4.5 years in these 42 patients. Utilization of the micro-axial LVAD in PCCS, with/without p-RVAD, can facilitate weaning from ECMO or even avoid postoperative ECMO completely, with its inherent complications [16]. This strategy greatly improves outcomes following high-risk cardiac surgery. 

In our series, p-RVAD support was required in 15 patients (15%). In the LVAD group, five patients required p-RVAD support. In the PCCS group, in conjunction with a micro-axial LVAD, 10 patients required p-RVAD + ECMO support not only for RV failure but also for oxygenation purposes. Interestingly, in the patients presenting with cardiogenic shock and undergoing placement of a micro-axial LVAD, either as a bridge to transplantation or as a bridge to a durable LVAD, mechanical offloading of the LV and consequent reduction in the LVEDP, in conjunction with inotropes, was sufficient to maintain RV function, without the requirement for a p-RVAD.

Prior studies utilizing these micro-axial ambulatory temporary LVADs have corroborated our results in group-1 and group-2 by demonstrating better unloading and recovery of LV function, shorter duration of ventilation, earlier ambulation, no ECMO-related complications, and therefore improved overall outcomes and survival [19,20,21].

Compared to previous studies [20], we were able to follow these patients for up to 4.5 years with good survival as outlined above. Furthermore, our study is probably the first to examine outcomes between patients undergoing placement of these devices in different categories of cardiogenic shock. Multivariate analysis demonstrated statistically significant 3.6–4.7-fold worse survival in the postcardiotomy support cardiogenic shock group compared to the group bridged to transplantation, utilizing the micro-axial LVAD as a bridging strategy to recovery from cardiogenic shock.

We found a higher incidence of stroke in our series compared to others [20]. However, due to our enhanced intensive care protocols, we were able to limit the extent of disability. The increased numbers of strokes were found in the PCCS category, who have greater pre-existing incidence of atherosclerotic and cerebrovascular disease. We developed a protocol of cutting the shaft of the M-LVAD following application of the aortic clamp during recipient cardiectomy during a transplant or through the ventriculotomy during implantation of the LVAD and removing the device directly this way, thereby eliminating the risk of dragging any clots on the device through the innominate artery. In patients where the device was removed several weeks following cardiotomy, we employed bilateral carotid occlusion along with occlusion of the distal axillary artery during withdrawal of the M-LVAD through the Dacron graft and performed proximal and distal embolectomies with an embolectomy catheter to flush out any residual clots. With these techniques, we were able to minimize the number of strokes (*n* = 2) in the second half of our cohort.

Future research is needed prospectively to compare the different MCS devices in cardiogenic shock patients particularly in the context of postcardiotomy which seems to result in a higher mortality. Additionally, the long-term quality of life and functional outcomes need further investigation. The use of tMCS in patients with cardiogenic shock as a bridge to definitive therapies is gaining importance in today’s practice of cardiology and cardiac surgery. Different devices may suit different clinical scenarios. One of the major goals for the future would be a focus on developing pumps utilizing artificial intelligence (AI) which could drive hemodynamic monitoring to predict potential danger and instability so that proactive treatment strategies can be adopted to improve survival and outcomes. Integration of AI-driven technology could pave the way for optimization of a combination of drug or device therapies. Such individualized treatment strategies will aid in accomplishing the goal of personalized medicine. Advancements in technology should be geared to optimize outcomes and minimize complications [22,23]. Integration of AI in temporary MCS may have a strong impact on revolutionizing patient management and outcomes. Use of AI-guided risk prediction models may be able to use real-time data from temporary devices to estimate cardiac function and predict patient outcomes and trajectories. Such technology could enhance the speed of decision making. Future research should also be directed at simulation of clinical scenarios to enable automation in choosing the right type of device for the right patient. Such approaches can also be used in managing hemocompatibility as well as early risk stratification of patients. The fine balance between bleeding and thrombotic complications drives optimal outcomes in MCS devices. Hence, optimal management of hemocompatibility is crucial for excellent outcomes [24]. Despite the challenges of using AI-driven technologies in the context of high-quality data and validation, AI still holds promise in clinical decision making and personalized medicine.

## 5. Limitations

The number of patients in the study was small. Furthermore, this was a single-center, observational, non-randomized study, which may introduce confounding variables and factors specific to the study site, and therefore the results may not be applicable to a wider population. However, it represents a clean group of patients operated on by a limited number of surgeons, with a consistent practice of M-LVAD placement in terms of indications, surgical technique, and selection of the appropriate triage category. Therefore, future research should involve larger, multicenter, randomized controlled trials to confirm the findings with greater generalizability and to serve as external validation of the data.

## 6. Conclusions

These temporary micro-axial intravascular LVADs have completely changed the way that cardiogenic shock has been managed over the last 4.5 years at our institution, with excellent mid-term outcomes. Future research should focus on comparing M-LVADs with other forms of mechanical circulatory support, such as ECMO, in more detail, particularly in the context of postcardiotomy CS where survival outcomes seem to differ significantly.

## Figures and Tables

**Table 1:** Preoperative characteristics by group.

Variable	Group-1 (*n* = 34)	Group-2 (*n* = 25)	Group-3 (*n* = 42)	*p*-Value
	Means ± SD	Means ± SD	Means ± SD	
Age (years)	53 ± 12.4	57.5 ± 13	63.3 ± 12.4	**0.002**
Body mass index	28.5 ± 3.9	29.1 ± 5.3	31.4 ± 6.1	**0.045**
	Number (%)	Number (%)	Number (%)	
Female	2 (5.9)	3 (12)	4 (9.5)	0.69
p-RVAD	0	5 (20)	10 (24)	**0.009**
ECMO to M-LVAD	3 (9)	6 (24)	11 (26)	**0.043**

SD: Standard deviation; ECMO: extra corporeal membrane oxygenator; p-RVAD: Percutaneous right ventricular assist device; M-LVAD: micro-axial left ventricular assist devices.

**Table 2:** Postoperative complications.

	Group-1 (*n* = 34)	Group-2 (*n* = 25)	Group-3 (*n* = 42)	*p*-Value
	Means ± SD	Means ± SD	Means ± SD	
Days on Impella support	27 ± 21	20 ± 14	14.5 ± 11	**0.003**
Intensive care unit days	38.7 ± 26	53 ± 30.5	27.8 ± 25	**0.002**
	Number (%)	Number (%)	Number (%)	
Axillary hematoma	5 (14.7)	3 (12.5)	2 (4.8)	0.32
Device malfunction	2 (5.9)	2 (8)	2 (4.8)	0.87
Gastrointestinal bleed	2 (5.9)	1 (4)	6 (14.3)	0.3
Stroke	1 (2.9)	2 (8)	8 (19)	0.07
p-RVAD	0	5 (20)	10 (27.8)	**0.004**
Dialysis	4 (11.8)	5 (20)	12 (28.6)	0.195
Hospital mortality	2 (5.9)	1 (4)	10 (23.8)	**0.021**

SD: Standard deviation; p-RVAD: Percutaneous right ventricular assist device.

**Table 3:** Logistic Regression: Predictors of hospital mortality.

	Odds Ratio (OR)	OR 95% Confidence Intervals	*p*-Value
p-RVAD	1.3	0.28–6	0.74
M-LVAD category	4.7	0.9–24	**0.05**

p-RVAD: Percutaneous right ventricular assist device; M-LVAD: Micro-axial left ventricular assist device.

**Table 4:** Cox regression multivariable predictors of long-term mortality.

Covariate	Hazard Ratio (HR)	HR 95% Confidence Interval	*p*-Value
M-LVAD category	3.63	1.03–12.9	**0.04**
Postoperative long-term hemodialysis	3.9	1.6–9	**0.002**
Gastrointestinal bleeding	1.5	0.5–4.5	0.43

M-LVAD: Micro-axial left ventricular assist device.

## Data Availability

All relevant data is included in the manuscript.

## Supplementary Materials

The following supporting information can be downloaded at: https://www.mdpi.com/article/10.3390/jcm13226804/s1, Video S1.
